# The Care of the Feet

**Published:** 1878-10

**Authors:** P. Harold Hayes


### THE CARE OF THE FEET.

P. HAROLD HAYES, M. D.

The exquisite natural sensibility of the feet, their nerve-associations with every organ of the body, and the healthy influences or morbid irritations, which may radiate therefrom, merit the most careful consideration. An exalted sense of comfort is felt from having the feet rubbed, or masseed by the warm hands of an attendant. This feeling of comfort pervades the entire body and has a most tranquilizing effect upon the whole nervous system. The same results are often felt in a large degree from the warm foot bath.

*Cold* feet are both the cause and the consequence of disease. In many diseases the heart's impulse is too feeble to send the pulse wave with any normal degree of force to the feet, or even to the hands. They will of course remain habitually cold. In many other diseases there are congestions of the brain, lungs, or abdominal organs, which divert the blood, and with it the warmth from the feet.

If the feet on the other hand are allowed to become cold from causes applied to themselves, they will in turn induce congestions, of any or all of the internal organs, bring on a general feeling of chilliness, restlessness and misery, specially suppressing the action of the skin, and preventing sleep. The best medical treatment will fail in every disease, acute or chronic, if the feet are allowed to remain cold.

Cold applied to the feet or hands is quickly felt at remote points. A piece of ice held in one hand will shortly lower the temperature of the other hand 15° and arrest a hemorrhage there. The operation for stone may be rendered almost bloodless, by immersing the feet of the patient in cold water. Cold applied to the feet at a critical time may not only arrest the menses, but the same influence may check the supply of arterial blood to all the organs concerned in digestion and nutrition, and so produce anemia and slow starvation, not for want of food, but for want of *blood* and *life* in the organs of digestion to appropriate it. Constipation is incident to this condition, though we have rarely thought of the feet having anything to do with this disease. Cold, especially cold and damp, to the feet can cause almost any known form of disease. It is a very common cause of *sciatica* with vaso-motor contractions of the arteries, diminished blood-supply, coldness and wasting of the affected limb, a small popliteal pulse, and intense pain. Lumbago, neuralgia, gastrodynia, jaundice, catarrh, heart disease and bronchitis are continually creeping into the system through cold feet. I have at this moment, under my professional care, a young lady who has not spoken a loud word in six weeks, and this condition of things followed the next morning after a barefoot frolic in the shallow water at the lake shore, of more than an hour's duration.

We have all had experience of the intense nervous sensibility of the feet from having had our feet tickled in childhood. But this sensation quickly becomes intolerable, and may excite disease. Van Sweiten says: I have seen a very healthy girl, of vigorous parents, rendered epileptic for many years, and seized with her first paroxysm while some girls tickled the soles of her feet in play, others holding her down, so she could not escape.

Corresponding with this acute sensibility of the feet it is found that no wounds are so dangerons as those in the sole of the foot. These wounds are sometimes followed by terrible neuralgia, and sometimes by that frightful disease known to the profession as *Tetanus*, and by the unprofessional called lock-jaw.

The reader must now be prepared to appreciate the care of the feet as a most prominent element of health and long life. One of the most common ways of taking cold through the feet, is by walking on the damp ground or pavement without the protection of rubbers, and then sitting in the school-room or in an office until the feet are thoroughly chilled. Rubbers themselves are a sort of necessary evil, for they are apt to overheat the feet and cause perspiration, and if too tight, they cause very cold feet by checking the circulation. Another and very insidious way of taking a mysterious cold is to drop off warm boots and put on light slippers.

Habitual cold feet are associated most generally with dyspepsia and poverty of the blood. Students and professional men have cold feet from too much use of the head and too little use of the heels. Cold feet intensify the very diseases that caused them, and will develop other diseases without number. I cannot recall at this moment a more striking illustration of a disease being at the same instant its own cause and its own effect. Cold feet are the *effect* of cold feet. Cold feet are the *cause* of cold feet.

The cure of cold feet is a double matter. Cure the disease upon which they depend. Cure the cold feet themselves or you cannot cure the disease which caused them. How? First, by dress. Wear loose boots, that the circulation may not be bound, and lamb's wool or merino stockings. If these make the feet perspire, put on fine cotton hose next the foot and light merino above. Exercise by walking is a most natural and radical cure for cold feet. Miss K—— was always complaining of cold feet. On inquiry, I found she was in the habit of poring over books much of her time, and when not thus engaged appeared to be in a sort of reverie. Was advised to walk the feet warm twice a day, and to engage in some handiwork and not to look in a book in the evening.

Many people who suffer habitually from cold feet have brought it about by the habit of sitting with the feet upon the stove or register. Others are constantly using hot bricks, hot flat irons or soapstones. These are sure ways of inducing a feeble circulation in the feet and inveterating the very suffering they seek to relieve. In bed, and for chronic invalids, a rubber bottle of hot water, not to exceed 110° Fahrenheit, may be permitted. It should be well covered with flannels.

The mustard and cayenne foot bath is a most efficient foot-warmer and a grand thing to use when we come home with feet cold and damp, perhaps with the head stuffed up and a feeling of tightness about the chest. Take two ounces of mustard and one ounce of cayenne pepper, put them into a pint bottle, fill with water and shake well, always before using. Of this preparation put a large tablespoonful into a gallon of hot water in a common pail. The feet may be kept in for ten to twenty minutes and a little hot water added now and then. When taken out they should be plunged into water about eighty degrees, and then wiped thoroughly dry. This foot bath is best taken when the patient is about to retire for the night. If taken at other times the patient should not go immediately out, but lounge or rest in doors, as it excites perspiration. I have had recourse to this foot bath in the chilly stage of ague, and in congestions of the brain and of the lungs with the most prompt and marvelous relief. When a very prompt and decided effect is desired, this foot bath should be made as deep as a common pail will allow, with a corresponding increase in the amount of the mustard and cayenne combination. When the perspiration following is profuse it should be rubbed off two or three times with dry and soft linen towels.

